# Inheritance of Acquired Behaviour Adaptations and Brain Gene Expression in Chickens

**DOI:** 10.1371/journal.pone.0006405

**Published:** 2009-07-28

**Authors:** Daniel Nätt, Niclas Lindqvist, Henrik Stranneheim, Joakim Lundeberg, Peter A. Torjesen, Per Jensen

**Affiliations:** 1 IFM Biology Division of Zoology, Linköping University Sweden, Linköping, Sweden; 2 School of Biotechnology, Department of Gene Technology, Royal Institute of Technology, Stockholm, Sweden; 3 Hormone Laboratory, Aker University Hospital HF, Oslo, Norway; University of Oxford, United Kingdom

## Abstract

**Background:**

Environmental challenges may affect both the exposed individuals and their offspring. We investigated possible adaptive aspects of such cross-generation transmissions, and hypothesized that chronic unpredictable food access would cause chickens to show a more conservative feeding strategy and to be more dominant, and that these adaptations would be transmitted to the offspring.

**Methodology/Principal Findings:**

Parents were raised in an unpredictable (UL) or in predictable diurnal light rhythm (PL, 12∶12 h light∶dark). In a foraging test, UL birds pecked more at freely available, rather than at hidden and more attractive food, compared to birds from the PL group. Female offspring of UL birds, raised in predictable light conditions without parental contact, showed a similar foraging behavior, differing from offspring of PL birds. Furthermore, adult offspring of UL birds performed more food pecks in a dominance test, showed a higher preference for high energy food, survived better, and were heavier than offspring of PL parents. Using cDNA microarrays, we found that the differential brain gene expression caused by the challenge was mirrored in the offspring. In particular, several immunoglobulin genes seemed to be affected similarly in both UL parents and their offspring. Estradiol levels were significantly higher in egg yolk from UL birds, suggesting one possible mechanism for these effects.

**Conclusions/Significance:**

Our findings suggest that unpredictable food access caused seemingly adaptive responses in feeding behavior, which may have been transmitted to the offspring by means of epigenetic mechanisms, including regulation of immune genes. This may have prepared the offspring for coping with an unpredictable environment.

## Introduction

Environmental challenges force animals to adjust their behaviour and physiology in order to cope. This affects their phenotype, and may also be associated with epigenetic modifications of gene expression patterns, both of which may be transmitted across generations. For example, lack of maternal care in rats causes a significant effect on brain gene expression and stress related behaviour later in life [1]. Similar effects may be seen in offspring exposed to elevated levels of steroid hormones, toxins or malnutrition *in utero* or *in ovo* [2]–[6]. Furthermore, we have previously shown that offspring of chickens raised in stressful conditions have an affected phenotype and brain gene expression which mirrors that of their parents [7], supporting other studies which have shown that offspring phenotypes may be affected by parental experiences preceding pregnancy, and even persist over more than one generation [8]–[10].

Studies on transgenerational effects are increasingly focusing on disease risks and stress related responses in the offspring [11]. However, it has been suggested that mechanisms such as these could be a means of preparing the offspring for a hostile environment [12], which, in principle, could be an adaptive mechanism, allowing offspring to increase their fitness when faced with similar challenges as those experienced by the parents.

An adaptive response should be relevant with respect to the type of challenge, and it should potentially increase the fitness of the affected individual [13]. Furthermore, an adaptive transgenerational effect would require that the specific response of the parents is mirrored in the offspring, making them better equipped to cope with the environmental challenges experienced by the parents.

In the present experiment, we investigated whether unpredictable access to food and water would cause adaptive modifications in behaviour and gene expression in chickens, and whether this would be mirrored in the offspring. Possible ways to adapt to such a situation would be, for example, to change foraging strategy towards feeding more at known food sites and spend less time exploring for new sites (a more conservative foraging strategy), to increase the rate of dominance related behaviors as a consequence of a higher competition for food with limited availability, and to aim for highest possible energy intake per time unit. Such changes in behavior could logically be presumed to increase the feeding efficiency in a situation where food availability is stochastic, and would therefore be expected to increase the fitness of an animal in such an environment. Optimal foraging theories have predicted similar shifts toward conservative strategies when animals are exposed to variable food availability [14].

Transmissions of information across generations which does not involve traditional inheritance of DNA-sequence alleles is often referred to as soft inheritance [15] or “Lamarckian inheritance”. It can broadly be divided into animal tradition and epigenetic inheritance (heritable changes in gene expression which occur without changes in nucleotide sequence) [16], [17]. Gene expression can potentially be affected across generations by means of various pathways: hormonal effects on embryos, effects of maternal behavior, and direct transmission of epigenetic marks such as methylation of DNA. Chickens are powerful model animals for studying epigenetic inheritance, since eggs can be incubated, hatched and offspring raised without any contacts with the parents, hence eliminating transmission of animal tradition between generations.

We hypothesized that in an environment with unpredictable food access, chickens will show more conservative and competitive foraging strategies, as well as increased preference for high-energy food. We further hypothesized that such alterations in behavior, and the associated modifications in brain gene expression profiles, would be transmitted to the offspring, thereby suggestively prepare the offspring for the parental environment.

To test these hypotheses we studied the behavior and brain gene expression in chickens raised in an unpredictable light rhythm (since chickens do not feed in darkness [18], this makes food availability stochastic), and their offspring, which were raised in a standard, predictable environment. We were able to show a significant transgenerational transmission of behavior, which seems to be adaptive in relation to the challenges experienced by the parents. Furthermore, using a 14000 transcript microarray, we found a correlation between the induced gene expression differences in the parents, and the corresponding difference between offspring of parents from unpredictable or predictable conditions. These results supported and further corroborated our previous findings, showing that adaptive responses can be transmitted across generations in chickens by means of epigenetic mechanisms.

## Results

We used two generations of a commercial line of chickens selected for high egg-laying. Parents were either raised from 26 days of age in a chronic unpredictable light/dark rhythm (UL-treatment; Unpredictable Light) or a predictable 12∶12 h light/dark rhythm (PL-treatment; Predictable Light). Since chickens do not eat in darkness [18], the UL treatment meant that also food availability was unpredictable. All offspring, both from UL and PL parents, were raised under predictable light.

**Figure 1:**
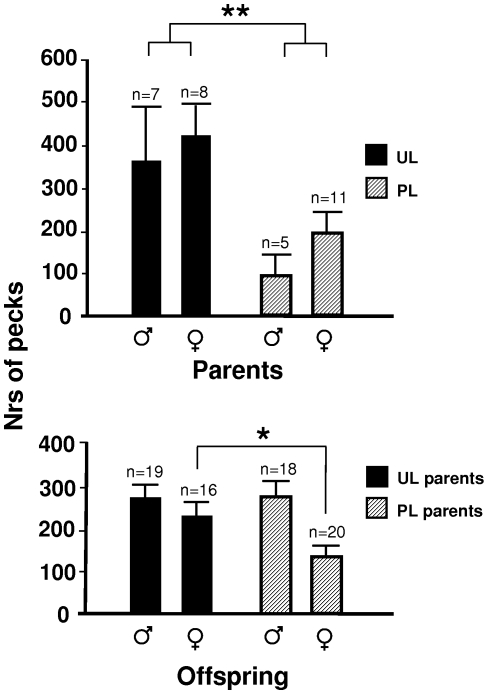
Average nrs of pecks±SEM directed to a familiar, readily available food resource in UL and PL parents, and their respective offspring, as measured in a Foraging test. Significant differences are indicated: * = p<0.05; ** = p<0.01.

Parents were firstly tested in a foraging test, where individual test birds each were allowed to feed in an arena containing three types of potential food sources placed in evenly distributed holes in the floor. One third of the holes contained freely accessible regular food and one third contained meal worms, a highly preferred food item to which the birds had earlier been accustomed. The meal worms were hidden in saw dust, so finding the food required searching and scratching. The last third contained only saw-dust (no food), so in effect, a bird had to choose between eating freely available standard feed or searching for a more unreliable, but attractive, food. UL parents showed an overall increase in the total number of pecks in the arena compared to PL (means±SEM: 595±63 versus 322±66 pecks, F_1,29_ = 7.3, p<0.01), and they pecked more in holes containing freely available regular feed (Figure 1).

**Figure 2:**
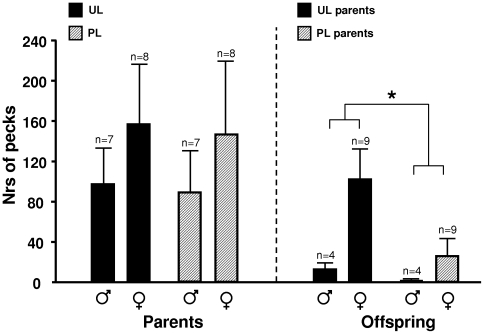
Food competition at adult age in UL and PL parents and their respective offspring as measured in a Dominance test. Numbers of pecks in the food are given in means±SEM. Significant differences are indicated: * = p<0.05.

**Figure 3:**
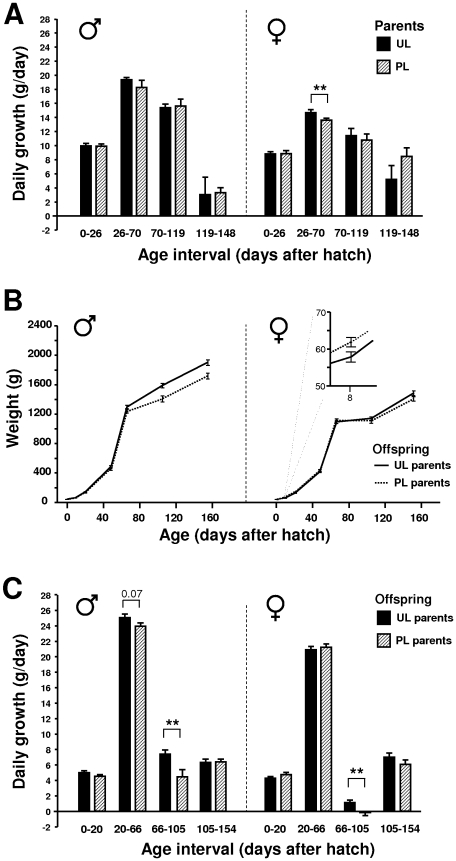
Preference for high energy food in offspring of UL and PL parents. Time spent close to the food sources is given in means±SEM. Significant differences are indicated: * = p<0.05. Dashed bars indicate preference for low energy food.

**Figure 4:**
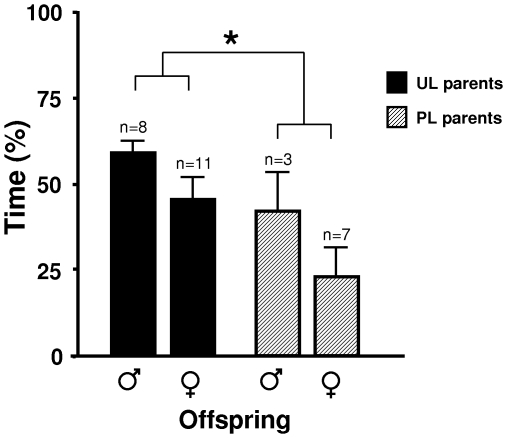
Weight measurements of surviving birds. (A) Growth in UL and PL parents; male UL, n = 4; male PL, n = 7; female UL, n = 11; female PL, n = 8. (B) Body weight in offspring of UL and PL parents. The difference between the offspring groups was significant (p<0.01) based on a repeated measure general linear model with parental treatment and sex as independent factors. There was an interaction between parental treatment and sex (p<0.05) illustrated by the small figure in the female diagram. (C) Growth in offspring of UL and PL parents; males with UL parents n = 10; males with PL parents, n = 9; females with UL parents n = 11; females with PL parents n = 10. All values are given as grams with means±SEM.

Secondly, the birds were exposed to a pair-wise dominance test, where two birds of the same sex, one from each treatment, competed for a common food resource which only one of them could access at the time. There was no effect of UL on the result in this test (Figure 2). Furthermore, there were no overall effects of UL on the weights at the different ages, but between day 26 and 70, right after onset of treatment in the UL group, UL parents had a higher growth rate than the PL (Figure 3); this was primarily due to an effect in the females,

Offspring, which had never been exposed to an unpredictable light rhythm, were also tested in a similar foraging arena and in a similar dominance test as their parents. Just like their parents, female (but not male) offspring of UL birds pecked more in food sources with freely available regular feed than the offspring of PL birds in the Foraging test (Figure 1). Unlike the parents, there was no treatment effect on the total number of pecks in the test. At adult age, but not when young, offspring of UL parents pecked more at the food in the Dominance test (Figure 2) and also showed shorter latencies to peck at the food (means±SEM: 199±68 versus 470±58 s, Z = −2.5, p = 0.01). In addition to these tests, the offspring were subjected to a food preference test, where they could choose between low energy and high energy food. Offspring of UL parents spent more time at the high energy food source, and less at the low energy food source (Figure 4). Offspring of UL parents were significantly heavier than offspring of PL parents; this was primarily due to a male difference late in life. There was also a significant interaction between parental treatment and offspring sex on weight (Figure 3). The interaction was due to female offspring of PL parents being significantly larger at day 8 (F_1,20_ = 4.5, p<0.05). When instead considering daily growth at different age intervals, both male and female offspring of UL parents grew significantly faster between day 66 and 105 than offspring of PL parents. In addition, offspring of UL parents had significantly higher survival at 40 weeks of age than offspring of PL (65% versus 39%, χ^2^ = 4.2, df = 1, p<0.05); similar effects of treatment was not seen in the parents.

When the parents were 20 weeks old, we collected eggs from UL and PL birds respectively, on the same morning (which reassured that each egg was laid by different females), and measured the levels of five steroid hormones in yolk and albumen. Only estradiol in yolk was significantly elevated by the UL treatment (1.04±0.04 mmol/l vs 0.87±0.04 mmol/l; F1,11 = 9.4, p<0.01) although the estradiol precursor testosterone had a non-significant tendency to be elevated as well (12.65±1.0 mmo/l vs 10.97±0.79 mmol/l).

RNA from the hypothalamus of eight birds from each treatment and generation was hybridized to 14k cDNA chicken microarrays in order to compare gene expression profiles between treatments and generations. In chickens, hypothalamus has previously been associated with stress regulation within and across generations [7]. Nine genes were significantly differentially expressed (DE) based on B-value (the log posterior odds ratio of differential expression vs non-differential expression; significance was set at B>0) between UL and PL in the parents (Table 1). A gene ontology analysis assigned three of the five annotated genes to a biological process (one gene per category): rhythmic process (corrected hypergeometric p<0.01), signal transduction (corrected hypergeometric p<0.05), and transcription (corrected hypergeometric p<0.05).

**Table 1:** Significantly differentially expressed gene transcripts between parents treated with Unpredictable Light or Predictable Light based on microarray analysis.

Symbol	Name	GenBank	UniGene	M-fold	B-value
	Unknown	CN237660		0.69	2.48
	Unknown	CN236687		0.87	2.45
*C1QC*	Complement component 1, q subcomponent, C chain	BU413814	Gga.9873	0.81	1.93
	Transcribed locus	CN227392	Gga.15444	0.51	1.78
*PER2*	Period homolog 2	BU311564	Gga.39390	0.73	1.54
*SOUL*	SOUL protein	CN225752	Gga.1806	−0.65	0.59
*CIRBP*	Cold inducible RNA binding protein	CN232810	Gga.4756	0.36	0.47
*CRIP2*	Cysteine-rich protein 2	BU123394	Gga.42630	0.78	0.31
*MAPK8IP3*	Mitogen-activated protein kinase 8 interacting protein 3	CN224884	Gga.16066	0.53	0.06

M-fold value is the log2 of the difference in expression level, and B-value is the log odds ratio of expression levels; the B-value estimates the certainty of DE vs non-certainty of DE. The criterium used for significant expression was that B>0. Positive M-values indicate that the transcript is higher expressed in birds treated with Unpredictable Light.

Considering the sexes separately, Heat shock 70kDa protein 5 (HSPA5, UniGeneID: Gga.4219) and Early growth response 1 (EGR1, UniGeneID: Gga.4922) were significantly down-regulated in UL males (B-value: 0.66 and 0.09 respectively) compared to PL. Based on the criterion B>0, no significantly DE genes were found in the offspring, although there were several genes with considerable fold-changes, i e, the estimated average expression levels were higher in one of the treatments.

**Figure 5:**
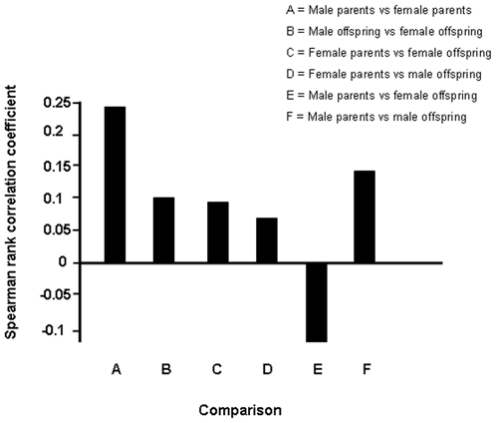
Correlations of differential gene expression. Spearman rank correlations between the M-values of the two sexes within and between generations. All correlations coefficients were significant on at least p<0.05.

To explore possible transgenerational effects in the altered gene expression caused by UL as compared to PL, we calculated the Spearman rank correlation coefficients between the M-values (fold change, measured as the log_2_ of the difference in expression level; indicator of the effects of treatment on the expression level of a gene) of the two sexes within and between generations (Figure 5). This analysis measures whether the UL-induced pattern of DE was similar in both sexes, and whether a similar difference could be found when comparing offspring of UL parents with offspring of PL parents. There were significant correlations (p<0.05) between male and female M-values within both generations, showing that male and female parents were affected similarly by the UL, and that this effect was mirrored, but to a lower degree, by a similar difference between offspring of UL and PL-parents. Across generations, the pattern of DE in both female and male offspring tended to mirror the UL-induced DE in the mothers, whereas only the DE of male offspring mirrored the fathers (all comparisons: p<0.05).

There could be concern that some of the effects in comparisons between males and females are due to dosage effects from the double Z-chromosomes of males. Adding the top 100 lists (based on M-values) of each sex and generation rendered a total of 114 fully annotated genes, and out of these, one of the parental male genes and one of the parental female DE genes were annotated to the Z-chromosome; in the offspring, one male and no female DE genes were annotated to the Z-chromosome. In total, the cDNA-array contained 7414 transcripts which have been assigned to a chromosome, and of these, 145 are situated on the Z-chromosome. Hence, in our data there was no over-representation of DE genes from the Z-chromosome (χ^2^ = 0.25; p = 0.61), excluding any possible suspicions about dosage effects in male-female comparisons.

Since a correlation pattern across generations was found, we compared the 2.5% top ranked genes (based on M-values of fold-change) of parents and offspring, in order to identify some of the genes particularly likely to show transgenerational transfer of DE, and Table 2 lists genes which occurred in the top lists of at least one of the sexes in both generations. Although care is needed when inferring function based on a list like this, it is notable that among the top rated DE genes in both generations were several transcripts from immune genes, which are separately shown in Table 3. Interestingly, three MHC-transcripts were among the top ranked in the parental generation, but not in the offspring.

**Table 2:** Genes with an M-value among the 2.5% top ranked in both generations, and sexes.

			Rank in parents		Rank in offspring	
Symbol	Name	UniGene	Female^A^	Male^B^	Female^C^	Male^D^
*ACSBG2*	Acyl-CoA synthetase bubblegum 2*	Gga.22498		213	86	
*SOUL*	SOUL protein	Gga.1806	174	56		8
	Immunoglobulin light chain C-region*/**	Gga.38	2	19	38	3
*MRPL19*	Mitochondrial ribosomal protein L19	Gga.9754		204		22
*LHX8*	LIM Homeobox	Gga.12515	12	NA	218	NA
*NFU1*	NFU1 iron-sulfur cluster scaffold homolog	Gga.22431		52	56	6
*LTF*	Lactotransferrin	Gga.2551	25			93
*CCNF*	Cyclin F	Gga.15366		1	1	30
*NUDT4*	Nudix-type motif 4	Gga.34977	58		57	13
*TTR*	Transthyretin	Gga.2620	18	22	109	1
*TMEM167B*	Transmembrane protein 167B	Gga.34569		46		25
*AHR*	Aryl hydrocarbon receptor	Gga.3264		18	26	NA
	Protein phosphatase 1, regulatory subunit 12C	Gga.30578	27		85	NA
*NCBP2*	Nuclear cap binding protein subunit 2	Gga.6356	57			119
*SCG5*	Secretogranin V	Gga.1678	86		43	
*FGFR3*	Fibroblast growth factor receptor 3	Gga.2908	30	191		74
*TSPAN15*	Tetraspanin 15	Gga.7579		80		2
	Immunoglobulin heavy chain */**		1	33	38	69
*BRPF3*	Bromodomain and PHD finger containing 3	Gga.7615	40			26
	Unkown transcript (GeneBank: CN220016)		8	34	54	5
*UBE2L3*	Ubiquitin-conjugating enzyme E2L 3	Gga.22647		91		11
	Transcribed locus	Gga.46489	56		75	
	Unkown transcript (GeneBank: CN223066)		163			58
*P2RY10*	Purinergic receptor P2Y, G-protein coupled, 10	Gga.25992	23	NA	6	NA
*ISG12-2*	Putative ISG12-2 protein	Gga.6201	NA	15	69	NA
	Unkown transcript (GeneBank: CN227633)			26	76	

The table shows the rank position of the M-value of a gene in a particular data set (for example, female parents). The highest rank is assigned to the gene with the highest absolute M-value, and the lowest possible M-value is equal to the total nrs of genes on the list after filtration. NA denotes genes filtered away during analysis as low quality spot on microarray. The criterion for a gene being listed in this table was that it belonged to the 2.5% highest ranked genes in at least one of the sexes in both generations.

* These genes were represented by more than one transcript on the microarray.

** All transcripts were top ranked in both generations, See table 3.

A Nrs of total genes on the list 9429.

B Nrs of total genes on the list 9570.

C Nrs of total genes on the list 9457.

D Nrs of total genes on the list 9078.

**Table 3:** Immune gene transcripts which had among the 2.5% highest absolute M-values in each sex and generation.

Name	GeneBank	UniGene	Parents			Offspring		
			Female	Male	All	Female	Male	All
Ig light chain	CN217151	Gga.22841	3		9		3	
Ig light chain	CN225453	Gga.22841	2	19	1		32	58
Ig light chain	CN224479	Gga.22841	5	49	2		134	141
Ig light chain	CN221461	Gga.22841	4		4	38	91	43
Ig heavy chain	CN234170	Gga.4330	104					
Ig heavy chain^1^	CN219711		6		22			
Ig heavy chain^1^	CN218849			33*		NA§	NA§	1
Ig heavy chain^1^	CN217762		1		7	92	69	36
MHC class I	CN224062	Gga.4973	150*		178*			
MHC class II	CN225201	Gga.4414	37	30	33			
MHC class II	CN221719	Gga.4414	31		199			

The table shows the same values with same criteria as in table 3 for all transcripts included on the microarray from the two immune related genes Ig light chain, Ig heavy chain. Also included are three MHC-transcripts, which were only top ranked in the parents. If not stated otherwise, all transcripts were down-regulated in parents treated with Unpredictable Light (UL) or offspring of UL parents in relation to parents in Predictable Light or their offspring.

* Up-regulated.

1 Annotation was done using Chicken Discovery System.

§ NA indicate genes filtered away during analysis as low quality spot on microarray. Filtration is dependent on joint properties on all the arrays in the group, hence could fall out differently in relation to which arrays are included.

Furthermore, we used the top 100 list of genes, based on M-values, in each generation and sex for a gene ontology (GO) analysis for biological processes. Only six parental genes (out of 59 annotated) were assigned a significant GO-function (one gene in each functional category), and these were T-cell differentiation, Positive regulation of JNK cascade, Epidermis development, B-cell activation, Notch signaling pathway, and Protein metabolic processes (all with corrected hypergeometric p<0.05). In the offspring, also six genes were assigned significant GO-functions, and these were Transmembrane receptor protein tyrosine kinase signaling pathway (3 genes; corrected hypergeometric p<0.01) and Regulation of transcription DNA-dependent (3 genes; corrected hypergeometric p<0.01). Hence, no GO-functions appeared as DE in both generations based on this analysis.

Five top ranked genes (two of which were significantly DE based on B-values in the parents) were selected for verification with real-time quantitative PCR: *Immunoglobulin light chain*, *LTF*, *SOUL*, *HSPA5* (Gga.4219) and *PER2*. In the parents, all genes were DE in the same direction as on the microarray, and four were significantly DE either on p-value (p<0.05) or fold change (>2 fold). This verification rate is in accordance with previous studies [19].

## Discussion

Our findings show that chickens in a stochastic light environment, which includes an unpredictable access to food, showed modification of their foraging behavior which is in line with what could be expected from an adaptive perspective. Furthermore, offspring of UL birds showed a similar response to that of their parents, in spite of themselves never being exposed to the treatment. As predicted, offspring of UL parents used a more conservative feeding strategy (females only), were more dominant and showed an increased preference for high energy food than offspring of PL parents. They were also heavier and had higher survival than the PL. The modified brain gene expression induced by UL was mirrored in the offspring, indicating a transgenerational transfer of the acquired differential expression levels. In addition, we found elevated estradiol levels in the yolk of eggs from UL hens, which may be part of the mechanisms involved in the transgenerational effect observed.

As predicted, the UL parents consumed more of the readily available food in the Foraging test, which can be interpreted as a more conservative feeding strategy. At the same time, they had a higher overall feeding rate, probably at the expense of explorative behavior. This illustrates a seemingly adaptive response to the challenge used in this experiment, where it would appear to be most beneficial to adopt the strategy of feeding as efficiently as possible at those unpredictable time windows when food is available. Furthermore, the increased growth rate, which was shown as an acute response to the onset of the treatment, is in agreement with previous reports [20], [21], and may indicate a general increase in feed intake in the home pens as a response to a sudden stochasticity of food availability.

Female offspring had a similar response in the same Foraging test as their parents, showing that the modified foraging strategy was transmitted to them. Additionally, the offspring of UL parents preferred high energy food, which again was in line with predictions, suggesting that the foraging strategy of the chicks was affected in an adaptive fashion by the UL experiences of the parents. This altered foraging behavior, possibly combined with an increased food intake, may partly explain the significantly higher weight gain in the offspring of UL birds.

Although no significantly DE genes were detected between the offspring of UL and PL parents based on B-values, the measured fold-changes of the expression of several genes and transcripts between UL and PL birds were closely related between generations. The correlation analysis, and the number of genes which appeared on the top ranked DE list in both generations suggests that the expression differences induced by UL in the parents were to some extent transferred to the offspring – if the expression of a particular gene was highly affected by the UL, the same gene tended to be similarly affected in the offspring of UL parents. Transthyretin and one of the unknown transcripts (GeneBank: CN220016) were of particular interest, because they were high ranked (within 1.2% of top ranked) in both sexes in both generations. Even though the Gene Ontology did not demonstrate any significant transgenerational effects on GO functional groups, it should be remembered that only relatively few genes were GO annotated. However, it is conceivable that some of the GO groups identified (although with small gene numbers) appear relevant in relation to the treatment and our results: for example, rhythmic process, transcription, T-cell differentiation and B-cell activation.

It should be noted that we have measured the gene expression at different ages in the parents and the offspring. This may have obscured the results, since it can not be excluded that some of the genes which are DE in the parents are not sufficiently expressed in the offspring at the age when we obtained our samples, and we may therefore miss some significant correlations. Unfortunately, the reference design we have used for the microarray analysis does not allow us to check whether a particular gene is expressed or not, since we only obtain the expression level relative to the reference sample. However, in a parallel experiment, where both parents and offspring were at the same ages (but a different White Leghorn breed) as in the present experiment and the same brain regions were studied, we used Affymetrix oligo-arrays (unpublished data). These do not use the reference design, and it is possible to estimate whether genes (or rather probe sets) have a detectable level of expression or not. The Affymetrix microarray contained 38535 probes, and using a signal strength of 5 as limit (considered to be just above background levels), 23153 had a detectable level in the parents, and 23037 in the offspring. Of these, 22778 were detectable in both groups. Changing the detection level to 7 decreased the number of detectable signals, but did not change the proportions. Although we can still not exclude that certain genes central to our experiment change their expression levels with age, we feel sufficiently safe in assuming that this should not alter our main findings. Regardless of the age differences between parents and offspring, our data strongly suggest that some DE was transferred between generations.

The correlation analysis suggests that the mother and father influenced offspring gene expression differently, maybe by parental imprinting on specific genes [22]. In the offspring, we found some differences between the sexes both in behavior and the possibly transmitted gene expression in response to the parental treatment. This is in accordance with findings by Mueller and Bale [23] who showed that the sexes were affected differently regarding emotionality after exposure to prenatal stress during pregnancy in mice and in their study, this difference was probably mediated by the methylation status of the corticotrophin-releasing factor and glucocorticoid receptor genes.

The microarray data from the parents suggest a possible coregulation of the expression of *PER2*, a clock gene which uses food occurrence as a zeitgeber [24], and some stress/immuno-related genes. Especially transcripts of the immunoglobulin light chain and its precursors were down-regulated in UL parents and tended to be so in their offspring. Other proteins of the immunoglobulin superfamily, like MHC class I and Thy-1, do not only contribute to different disease resistance phenotypes, but also have an apparent role in neural development [25]–[27]. It remains an intriguing possibility for future studies that immune genes, which give rise to extreme variation in gene products and play an important role in the complexity of neural development, also are involved in transgenerational epigenetic phenotypic tuning. If so, the impact on our understanding of the developmental origin of health and disease [28], [29] could be significant.

Since we have effectively excluded animal traditions in this study, transgenerational epigenetic inheritance is the most likely explanation to the differences seen in the offspring phenotypes. Epigenetic inheritance can be either context-dependent or germline dependent [30]. Context dependent epigenetics involve modifications in gene expression patterns caused by variations in the embryonic chemical and endocrine environment, where typical mediators are steroid hormones [31]. Germ-line dependent epigenetics, on the other hand, are mediated by meiotic and embryonic survival of epigenetic modifications in germ cells, like DNA-methylation or variations in chromatin structure [32]. An important difference between context dependent and germ-line dependent effects is that the former only shows a maternal effect, while the latter can originate from both parents [33]. A detailed analysis of the relative contribution of maternal and germ-line effects would require a different experimental design, where the parents of different sex were treated independently. Although this was not the immediate purpose of the present experiment, we have nevertheless performed a number of analyses to provide a first indication of possible transgenerational pathways.

The higher levels of estradiol in yolk of UL might have mediated the transgenerational effect in a context dependent manner. For example, maternal estradiol has been shown to affect embryonic brain development leading to increased anabolism and masculinized behaviors [34], much in agreement with the phenotypic effects we observed in the offspring. Furthermore, steroids are potent modifiers of gene expression [35] but it remains to be investigated how a particular steroid would be able to affect a specific subset of genes, as would be required in order to explain the relation between DE of specific genes in the two generations. Sex hormone deposits in the yolk come mainly from the ovaries, which appear to deposit egg hormone levels independently from the circulating levels [36], hence offering a mechanism for a female bird to change offspring phenotypes without affecting her own.

Similar to our previous results [7], corticosterone levels in eggs were not affected by the UL treatment. Since steroid levels in albumen reflect circulating levels in the mother [37], this indicates that the chronic unpredictable light rhythm at the most exerted a mild stress on the female birds at the time when eggs were collected.

The fact that we found significant correlations between DE in male parents and their male offspring may indicate that other mechanisms than hormonal effects via the egg might also play a role in the transgenerational transfer. Although the nature of such mechanisms remains to be discovered, similar effects have been observed in studies on humans [9].

Although there are many possible non-adaptive explanations for our results which we have not tested in this experiment, it remains an intriguing possibility that responses to environmental challenges may serve an important role in rapid adaptation across generations. Epigenetic modifications are normally transitory in their nature, but have been demonstrated to sometimes remain stable over several generations [15]. Transgenerational effects may aid in rapid evolutionary radiation [38], for example during major evolutionary transitions such as in bottleneck populations, domestication or in populations exposed to climate change or geographical isolation. This opens a new perspective on the communication between the environment and the genome.

## Materials and Methods

### Ethical considerations

This study was approved by the local Ethical Committee of The Swedish National Board of Laboratory Animals. The committee has the task to evaluate the welfare of the animals in relation to the purpose of the study, possibilities to alternative methods and to determine if the study is a repetition of an already conducted experiment.

### Animals and housing

A commercial laying hen hybrid, HyLine W98, was used in this experiment. Eggs were purchased from a commercial breeder and incubated in one and the same incubator (Masalles Comericial SA, Incubator type 25 H). The hatched chicks were marked, vaccinated and housed in 100 (w)×200 (l)×180 (h) cm pens, with ad libitum food and water, on a 12∶12 h light/dark rhythm. At day 26 after hatching 40 chicks were randomly selected, divided into two equally large groups, which were balanced for sex and weight, and introduced into two larger pens, each measuring 150×300×270 cm. As in Lindqvist et al. [7], one group was given an unpredictable light schedule (UL), while the other remained on a predictable 12∶12 h light∶dark cycle (PL). For the UL group light and dark periods of 3, 6, 9, 12, 18 and 24 hours were randomly applied. On a weekly basis, the light to dark ratio was balanced so that the total number of light hours per week was identical for the treatments. The two groups were housed in the same room and to minimize pen effects every third week the groups were moved between pens. Parents were weighed at day 0, 9, 26, 70, 119 and 148 after hatch. From six weeks of age both groups were offered meal worms three times per week to get accustomed to this food type. The mealworms were always presented in the same type of bowl as was later used in the Foraging test (see below).

At 24 weeks of age eggs were collected from each group once every morning and stored at 14°C. Observations showed that at least 60% of the males were actively copulating during this time, and daily egg counts showed that all females were contributing to the next generation (UL: max 9 eggs/day, mean 4.8±0.5 S.E.M.; PL max 9 eggs/day, mean 5.4±0.1 S.E.M). All eggs were incubated in the same incubator. In total 78 offspring were hatched and housed, as one group, in the same type of pens as the parents prior to onset of the treatment; 39 of those had UL parents and 39 had parents of the PL group. At an age of 26 days, they were moved to 300×300×180 cm home-pens, with perches, nest boxes, and *ad libitum* food and water. All offspring had a 12∶12 h light schedule, were presented meal worms once every second day and were weighed at day 0, 8, 20, 47, 66, 105, 154, 185 and 283 after hatch. Parents and offspring were housed in different buildings more than 100 km apart.

### Behavioral tests

The behavior of the birds was recorded in a series of tests designed to measure the hypothesized adaptive changes to the unpredictable light schedule (increased conservative foraging strategy, dominance and preference for energetic food).

### Foraging test – Conservative foraging strategy

To investigate foraging strategy all parental animals were on day 131-133 after hatching tested in a circular shaped (Ø = 250 cm) foraging arena. The floor of the arena had twelve holes (Ø = 10 cm), where the test bird had free access to three different resources, presented in equal numbers and balanced for spatial distribution: 1) familiar/regular food, 2) meal worms mixed with wood shavings, and 3) only wood shavings. A 50×50 cm start-box was connected to the arena with a guillotine door. Recordings were done with the behavioral sampling software The Observer 5.0 (Noldus Information Technology) through three video cameras monitoring the whole arena.

One day before the test all birds were habituated to the arena for 2×20 min in familiar groups of four individuals. At the test day birds were caught in their home pen and taken to a treatment specific pre-test pen, with *ad libitum* water, but without food (150×150×270 cm). After 120 min (±15 min) test birds were caught in the pre-test pen and in darkness introduced to the start-box. When light was turned on the birds were given 30 sec to calm down, followed by the lifting of the guillotine door, which gave the bird access to the whole arena and denoted the start of the test. One third of each treatment group was tested each day during the three day test period. By shifting the position of the three resources each day, the spatial occurrence of every food source was balanced in relation to treatment and start-box position. Observation was performed during 15 min and number of pecks to each of the resources was recorded.

The offspring was tested on day 55–57 after hatching in the same way as their parents in a similar, but smaller arena (Ø 120 cm), which was monitored with only one video camera.

### Dominance test

On day 146 each UL parent was tested together with a PL matched for sex and body weight. In darkness each pair was introduced to a 150×150×270 cm pen, with a perch, *ad libitum* water, but no food. After a habituation period of one hour light was turned off again, a familiar food container with regular pellets was introduced, followed by light and a 7 min observation period. The container allowed only one bird at a time to eat from it and the number of pecks directed toward food in the container was recorded.

All surviving offspring was tested at day 22 and 189 after hatch in the same way as the parents with the following exception: for the 22 days old chicks we used a smaller test pen (80×40×40 cm) with no perch. All recordings were done using The Observer 5.0 (Noldus Information Technology) behavioral sampling software.

### Food choice test (offspring only) – Preference for energetic food

On day 216 all surviving offspring were tested in a food preference test, using an equal sided triangular-shaped arena (150×130×200 cm) with different food sources in two corners; 1) a food container with low energy pellet (fat content: 3.5%); 2) a food container with high energy sunflower seeds (fat content: 49.4%); the third corner that was used as starting point for the test bird. Birds were food deprived for 2 h before the test. The 10 min test was started by lifting a bird into the arena, in complete darkness, and turning on the light. The location of the test bird was sampled automatically from, a video image with behavioral software Ethovision version 2.1.6 (Noldus Information Technology), where two parallel arenas were monitored simultaneously. Birds that did more than three flight attempts were excluded from the analysis due to problems with software recordings (in total one with UL parents and two from PL). The variables sampled were time spent in a zone close to each of the two food sources. Direct observations, which were not quantified, asserted that pecking was performed only when the birds were present in the zone.

### Behavioral statistical analysis

All behavioral analysis was done with the statistical software SPSS version 15.0 using General Linear Models (GLM), except for the Dominance test where a pair wise Wilcoxon signed ranks test was used. All data used in the GLM's were tested for normality of the standardized residuals (Shapiro-Wilkins test) and equal variance (Levene's test). Transformation was done on the data that did not reach the criteria for the GLM. Analysis of treatment-effects that included groups with mixed sexes always used both sex and treatment as independent factors.

### Survival rate

Differences in survival between treatments was conducted at 40 weeks of age using a chi-square test.

### Analysis of steroid concentrations in eggs

In the morning of day 138 after hatching six eggs from the UL and seven from the PL group was collected. From these, approximately 10 ml of yolk and albumen were frozen to −70°C, and later analyzed for testosterone, estradiol, androstendion, corticosterone and dihydrotestosterone.

Extraction from yolk was performed essentially as described by Schwabl [39] with some modifications. Initial extraction was performed with 10 ml diethyl ether, and recovery controlled by the addition of tritiated DHT or androstendione. The final extracts were dissolved in 2 ml 2,2,4-trimethylpentane. 1 ml was used for DHT assay after celite column purification. 0.2 ml was used for direct determination of androstendione and 0,5 ml for direct determination of corticosterone. The final organic phases were dried under nitrogen, and the dried extracts redissolved in 0,1% gelatine in PBS for RIA of corticosterone and androstendione. Another extract was made for the assay of estradiol and testosterone, where the final dried extracts were dissolved in 1 ml of diethylether which was divided into 2 parts that were separately dried under nitrogen. The 2 dried extracts were dissolved in the appropriate assaybuffers for immunoassay determinations.

Extraction of albumen was performed with 0.5 ml albumen in 0.5 ml distilled water, mixed and extracted with 7 ml of diethylether. After freezing, the ether phase was collected, dried under nitrogen and the residue dissolved in the appropriate assaybuffers.

The following RIAs were used for the quantification of the steroids: Corticosterone – as described by Lindqvist [7]; Testosterone – RIA kit from Orion Diagnostica, Espoo, Finland; Estradiol – DELFIA kit from PerkinElmer Life Sciences, Wallac Oy, Turku, Finland; Androstendione and DHT – as described by Opstad & Aakvaag [40].

### Brain sampling and RNA isolation

At day 179 after hatch 16 parents (8 males/8 females; 8 UL/8 PL) where sacrificed by decapitation. Brains were removed and the hypothalamus and pituitary region dissected as been described previously [7]. The time from sacrifice to snap freezing in liquid nitrogen was less then 15 min. The tissue collection of the offspring was performed with the same protocol but at day 58 after hatch. Samples were later transferred and stored in a −70°C freezer.

Prior to RNA isolation each sample was weighed frozen in 50 ml tubes. Appropriate amount (1 ml/50 mg) of TRI reagents (Ambion Inc.) was added and homogenized with a handheld motorized Ultra Turrax T8 homogenizer (IKA®). The samples were divided into 1 ml fractions and centrifuged for 10 min at 12000 x g (4°C). Supernatant was saved and 100 µl of 1-brom-3-chloropropane (Aldrich®) added. Incubation was performed for 5 min (20°C), followed by centrifugation for 15 min at 12000 x g (4°C). RNA was precipitated from the aqueous phase by adding 0.5 ml isopropanol, incubated for 10 min (20°C) and centrifuged for 10 min at 12000 x g (4°C). The supernatant was discarded and the pellet was washed in 1 ml 75% RNase free EtOH. After centrifugation for 10 min at 7500 x g (4°C), the pellet was dried and then re-dissolved in 30 µl RNase free H_2_O (Ambion Inc.).

RNA quantity and quality was assessed using a NanoDrop® spectrophotometer (NanoDrop Technologies Inc.) and the Agilent 2100 Bioanalyzer (Agilent Technologies). RNA integrity numbers never dropped under 8.0.

### Synthesis of cDNA and Microarray hybridization

For more information about the microarrays and protocols, see www.ktharray.se (protocol SOP 002 and 003). Production of cDNA, Cy3/Cy5 labeling and hybridization procedures have been described elsewhere [7], [41]. In short, 20 µg of total RNA was used for producing labeled cDNA, which was hybridized to the KTH UniChicken 2×14k cDNA microarray. The microarray, developed by the Royal Institute of Technology in Stockholm, contains 12.7k unique transcripts and was the same microarray as used in previous similar studies, allowing some comparison across experiments [7]. The microarray was constructed from an EST-library of chicken testes and brain, and details of the construction and content of the microarray has been described elsewhere [44]. Each individual test sample (Cy5) was hybridized against a differently labeled reference (Cy3), which was constructed by pooling total RNA-isolates from the hypothalamus/pituitary of 49 birds from two different breeds (including the samples of the present study). Scanning was performed by a MicroArray Scanner G2565B (Agilent Technologies, Serial nr US22502515).

### Microarray analysis

Raw fluorescence scan-data was imported to the software GenePix (Molecular Device Corp.), where the Cy5 and Cy3 channels were superimposed upon each other, creating spot colors ranging from red (sample overrepresentation), to yellow (equal representation), to green (reference overrepresentation). Spot identification and manual flagging of poor quality spots was also performed in GenePix, with an operator that was blinded for treatment, and transcript name and function. Pre-normalization quality control was performed by inspection of density plots in accordance with the protocol, in order to reassure that no aberrant arrays were included in the analysis.

For filtration, normalization and statistical analysis the R software environment with version 2.2.0 was used with the KTH-package add-in, using the same methods as detailed in Lindqvist et al. [7]. The strictest filtration criteria were used with the following commands in R: filterFlags, filterSize, filterB2SD, filterMtoM, filterRatioComp and filterSaturated. After filtration, approximately 60% of the total spots remained. Print-tip lowess normalization was performed within slide and spot intensities were normalized across slides.

The fold changes, given as M-values (the log2 of the difference in expression level),were calculated for each spot as in Lindqvist et al. [7], while the B-test was calculated as in Rubin et al. [41]. The B-value is attained by Bayesian statistics and assigns the log posterior odds ratio of differential expression versus non-differential expression. Previously the threshold for significance, taking false discovery threshold into account, has been set to B>0 for this type of tissue and microarray [7], [19], [41]. Spearman rank correlations of M-values (regardless of level) were calculated on all spots, comparing within and between sexes both within and between generations. Annotation was primarily done with NCBI's Entrez UniGene (http://www.ncbi.nlm.nih.gov/), and secondarily with Chicken Discovery System (http://www.sbc.su.se/~arve/chicken/).

Gene ontology analysis was performed using GeneCodis 2.0 (http://genecodis.dacya.ucm.es/), which calculates significant associations of genes to different ontological groups using hypergenometric distribution statistics with adjustment for false discovery rates.

### q-RT-PCR verification

Primers for ten high ranked genes differentially expressed were designed using Primer 3 [42]. When possible, all primer pairs were designed to span over exon-exon boundaries and a blast against the chicken genome (www.ensembl.org/Gallus_gallus) was performed to verify sequence uniqueness.

Isolated RNA samples were converted to cDNA in 50 µl fractions using TaqMan® Reverse Transcription reagents (Applied Biosystems). Before reverse transcription 17.25 µl of diluted RNA (1 µg) was treated with 2 µl Turbo DNase (1 U/µl) (Ambion/Applied Biosystems) in 5 µl 10× TaqMan RT buffer. Each sample was then heated to 37°C for 30 min, followed by 75°C for 7 min. The proceeding protocol was according to the manufacturer's recommendations. Quantitative real-time PCR was performed in 25 µl fractions in single plex on a Rotorgene® 6000 (Corbett Life Science), using Power SYBR-green reagents (Applied Biosystems), with each well containing: 12.5 µl 2× Power SYBR-green Master Mix, 1.5 µl of each primer (2.5 pmol/µl) and 4.5 µl RNase free water (Ambion/Applied Biosystems). The fold change and p-values was calculated with REST-386 excel-macro using the Pair Wise Fixed Reallocation Randomization Test with 2000 permutations and with three reference genes (*GAPDH*, *Beta-Actin* and *TATA-box binding protein*) [43].

### Supporting Information

#### Accession Numbers

The microarray experiment, described according to MIAME guidelines, has been deposited in ArrayExpress microarray data repository (http://www.ebi.ac.uk/microarray-as/ae/). Accession number is pending.
