# Correction: Inheritance of Acquired Behaviour Adaptations and Brain Gene Expression in Chickens

**DOI:** 10.1371/annotation/4f90ac09-ae5e-469a-a2f3-21a5ac68dc31

**Published:** 2009-08-21

**Authors:** Daniel Nätt, Niclas Lindqvist, Henrik Stranneheim, Joakim Lundeberg, Peter A. Torjesen, Per Jensen

The legends for Figures 3 and 4 were incorrectly switched. The correct legend for Figure 3 should read: "(A) Growth in UL and PL parents; male UL, n = 4; male PL, n = 7; female UL, n = 11; female PL, n = 8. (B) Body weight in offspring of UL and PL parents. The difference between the offspring groups was significant (p<0.01) based on a repeated measure general linear model with parental treatment and sex as independent factors. There was an interaction between parental treatment and sex (p<0.05) illustrated by the small figure in the female diagram. (C) Growth in offspring of UL and PL parents; males with UL parents n = 10; males with PL parents, n = 9; females with UL parents n = 11; females with PL parents n = 10. All values are given as grams with means±SEM."

